# Sarkosyl differentially solubilizes patient-derived alpha-synuclein fibril strains

**DOI:** 10.3389/fmolb.2023.1177556

**Published:** 2023-08-09

**Authors:** Hjalte Gram, Vasileios Theologidis, Thomas Boesen, Poul Henning Jensen

**Affiliations:** ^1^ Danish Research Institute of Translational Neuroscience—DANDRITE, Aarhus University, Aarhus, Denmark; ^2^ Department of Biomedicine, Aarhus University, Aarhus, Denmark; ^3^ Department of Nuclear Medicine and PET, Aarhus University Hospital, Aarhus, Denmark; ^4^ Interdisciplinary Nanoscience Center (iNANO), Aarhus University, Aarhus, Denmark; ^5^ Department of Molecular Biology and Genetics, Aarhus University, Aarhus, Denmark

**Keywords:** α-synuclein, aggregates, strains, sarkosyl, solubility, seeding

## Abstract

Insoluble α-synuclein (αSyn) filaments in brain tissue are a hallmark of Parkinson’s disease (PD) and Multiple system atrophy (MSA), and for structural studies, they have for decades been extracted using the detergent sarkosyl. We asked if PD and MSA patient-derived αSyn filament strains display different stability to sarkosyl extraction as this may confound our interpretation of the landscape of structural strains present in patients’ tissue. We compared the stability of cerebrospinal fluid-derived strains from four PD and four MSA patients using sedimentation and immunoassays and tested the seeding competence and strain-specific characteristics of the sarkosyl-soluble fractions using a seed amplification assay (SAA) and Thioflavin T (ThT) fluorescence. We demonstrate that filaments from PD are less resistant to sarkosyl than from MSA after they have been subjected to freezing and sonication. An enhanced release of monomers from PD filaments was the major difference between PD and MSA, but the sarkosyl-soluble fraction released from both PD and MSA filaments contained aggregates that displayed aggregate-specific epitopes and seeding activity with preserved disease-specific strain characteristics. Our results demonstrate that sarkosyl differentially destabilizes patient derived αSyn filament strains, which may compromise our ability to fully appreciate the landscape of αSyn filament currently being uncovered by high resolution cryoEM analyses. This should motivate an effort to develop more gentle extraction protocols.

## Introduction

Parkinson’s disease (PD) and multiple system atrophy (MSA) belong to the group of neurodegenerative diseases, synucleinopathies. They are characterized by the presence of cytoplasmic inclusions containing aggregates of the neuronal protein α-synuclein (αSyn) in affected brain cells. The inclusions in PD, so-called Lewy bodies, are located in neurons, while the inclusions in MSA are present as glial cytoplasmic inclusions in oligodendrocytes (Goedert et al., 2017).

The mechanisms that cause native αSyn to form aggregates in synucleinopathies are not well understood, but the process can result in the formation of both soluble oligomeric species and insoluble amyloid-type protofibrils that can assemble into double-helical filaments. The aggregation process can start by a slow *de novo* assembly of monomers into so-called seeds, at which point the seed can rapidly recruit monomers and form the filaments. The process can also be nucleated by introducing preformed aggregates that immediately seed rapid fibril growth.

αSyn aggregates are hypothesized to be prodegenerative as corroborated by inoculation of either preformed fibrils (PFF) or brain extracts containing αSyn seeds into the nervous system of mouse models where they templated the aggregation of endogenous αSyn resulting in neurodegeneration (Mougenot et al., 2011; Luk et al., 2012; 2013; Prusiner et al., 2015). There is a strong experimental link between the structure of the fibril and its functional impact, as *in vitro* generated structural variants of *de novo* formed fibrils cause different seeding properties and toxicity in cells and mice models (Bousset et al., 2013; Ferreira et al., 2021) and similar effects have been observed when inoculating animal models with αSyn aggregates isolated from human brain affected by synucleinopathies (Prusiner et al., 2015; Van der Perren et al., 2020).

The hypothesis that structural differences between different αSyn aggregate strains govern their different impact on cellular functions and ultimately patient symptoms, makes it imperative that we can isolate the entire repertoire of pathological αSyn aggregates for structural analyses. So far, soluble αSyn aggregate species isolated from brain tissue, collectively called oligomers, have not been the subject of detailed structural analysis. Instead, the focus has been on the insoluble filaments more easily purified by sedimentation protocols.

The successful development of cryoEM and structural reconstruction software has resulted in an increasing number of reports on high-resolution structures of protein aggregates isolated from common neurodegenerative diseases. This has allowed a structure-based classification of tauopathies (Shi et al., 2021). For synucleinopathies, it has demonstrated disease-specific filaments for DLB and MSA, and even the existence of different filaments structures in the same MSA brain (Schweighauser et al., 2020; Yang et al., 2022). The insoluble αSyn filaments isolated in sarkosyl-containing buffers from PD, DLB, and MSA brains were initially studied by negative staining transmission electron microscopy (EM) that gave important but limited structural insight, like straight vs. twisted fibrils (Grazia Spillantini et al., 1998; Spillantini et al., 1998).

Most current protocols for extracting ⍺Syn fibrils are based on homogenizing brain tissue in a buffer containing the non-denaturing detergent N-Lauroylsarcosine (sarkosyl) followed by centrifugation steps (Peng et al., 2018; Lau et al., 2020; Schweighauser et al., 2020). Here the insoluble fibrils are generally considered stable in sarkosyl, and the pelleted fibrils thus representative of all αSyn fibrils in the tissue. We demonstrate that insoluble αSyn filament strains derived from αSyn seeds in CSF of PD and MSA patients exhibit differential stability in sarkosyl, and the soluble αSyn species released from the filaments retain strain-specific seeding activity. This differential solubility of αSyn filaments in sarkosyl raises the concern that the current extraction procedures cannot isolate the full spectrum of αSyn filaments when producing material for functional and high-resolution cryoEM studies and warrants further optimization of gentler protocols.

## Materials and methods

### Patient-derived PFF generation

The PD and MSA patient-derived PFF were a generous gift from Mohammad Shahnawaz and Claudio Soto. Their characterization and detailed protocol have been previously described (Shahnawaz et al., 2020). In brief, they performed SAA using 1 mg/mL αSyn in 100 mM PIPES, pH 6.5, and 500 mM NaCl at a final volume of 200 μL seeded with 40 μL patient CSF. We received products of the finalized SAA and then performed amplification cycles to increase the PFF yield. Amplification was performed by reconstituting lyophilized wt monomeric αSyn in PBS pH 7.4 (Gibco). The monomer was then filtered through a 100 kDa filter to remove unwarranted oligomer species and then adjusted to 1 mg/mL. The SAA product was sonicated with a Branson SFX 250 (30 ms on, 70 ms off, 6 min total on, 30% power) and used to seed the monomer solution at 5% (m/m). The reaction was incubated at 37°C with continuous shaking at 1,050 rpm for 72 h. The generated PFF were harvested by centrifugation at 15,600 g at 25°C for 30 min and then resuspended in PBS. This amplification was repeated three times, using the product to seed the following amplification. The PFF were frozen in aliquots at −80°C until use.

### αSyn/p25α PFF generation

αSyn/p25α were generated as previously described (Ferreira et al., 2021). In brief, 346 μM soluble monomeric wild-type αSyn were incubated at 37°C in the absence or presence of p25α (17 μM) in PBS pH 7.4 (Gibco) with continuous shaking at 1,050 rpm (Eppendorf Thermotop) for 72 h. The generated PFF were harvested by centrifugation 15,600 g at 25°C for 30 min and then resuspended in PBS to a concentration of 2 mg/mL, as determined by BCA protein concentration assay (Pierce) using 0.1 M NaOH as diluent to dissociate the PFF completely. The PFF were frozen in aliquots at −80°C until use.

### Sedimentation assay

PFF (0.24 mg/mL) was incubated in PBS (10 mM phosphate, 123 mM NaCl, pH 6.6) with or without 1% sarkosyl (Sigma-Aldrich), 1% Triton X-100 (AppliChem), 1% CHAPS (Calbiochem) RT for 30 min. The samples were then centrifuged at 25.000 x g for 30 min (1.5 mL microcentrifuge tubes, Eppendorf 5417R centrifuge), and the supernatant and pellet fractions were separated. The pellet fraction was resuspended in PBS to the original volume. SDS sample buffer (100 mM Tris, pH 6.8, 40 mM DTE, 8% SDS, 24% glycerol, 0.02% bromophenol blue) was added to pellet- and supernatant-fractions 1:3, which were heated to 96°C for 15 min. Equal volumes of supernatant and pellet fraction were separated by SDS-PAGE using 8%–16% polyacrylamide gels (GenScript). Gels were scanned and quantified using ImageJ software (National Institutes of Health, Bethesda, MD, United States).

### Thioflavin T and K114 fluorometry

Amyloid-binding dyes thioflavin T (ThT) and (trans, trans)-1-bromo-2,5-bis-(4-hydroxy) styrylbenzene (K114) were used to assay the presence of amyloid structure in the PFF. Samples were analyzed by incubating PFF at 0.04 mg/mL in 90 mM glycine, pH 8.5, and either 20 µM ThT or 50 µM K114 for 15min. Fluorescence was measured with an EnSpire 2,300 Multilabel Reader (Perkin Elmer) at λ_ex_/λ_em_ = 450 nm/482 nm for ThT and λ_ex_/λ_em_ = 380 nm/550 nm for K114.

### Sonication and dynamic light scattering (DLS)

PFF was subjected to ultrasonic breakage using a Branson SFX 250 sonifier with a water-cooled cup horn attachment (30 ms on, 70 ms off, 6 min total on, 30% power). DLS measured the hydrodynamic size of the PFF fragments with a Wyatt DynaPro NanoStar instrument at 25°C. Ten measurements of 5 s each were averaged for each histogram, and this was done four times to ensure correct measurements. Data were analyzed with Dynamics V7.5.0.17 software using manufacturers’ pre-set solvent properties of PBS.

### Dot-blot

Samples from sedimentation assay diluted 1:500 in PBS, and monomeric ⍺Syn (100 ng, 50 ng, 25 ng, 10 ng, and 5 ng) were applied directly onto a 0.45 µm pore size nitrocellulose membrane using a vacuum filtration system (Bio-Rad BioDot Apparatus). Membranes were then briefly washed in TBS/T and blocked for 1 h at RT in 5% nonfat dry milk in TBS/T. Membranes were then incubated at 4^o^C ON with primary antibodies anti-αSyn Syn-1 (BD Biosciences, 1:1,000) or aggregate-specific MJF14-6-4-2 (MJF14) (Abcam ab209538, 1:450,00) and subsequently incubated with secondary horseradish peroxidase-conjugated anti-mouse (Dako) for 1 h at RT. Protein dots were visualized using ECL in a Fuji LAS-3000 Intelligent Dark Box (Fujifilm, Japan).

### Seed amplification assay (SAA)

PFF was incubated at 1 mg/mL in 1% sarkosyl in PBS (10 mM phosphate, 123 mM NaCl, pH 6.6) for 30 min at RT. To ensure complete sedimentation of sarkosyl-treated samples, samples were ultracentrifuged at 300,000 x g for 1 h. Furthermore, only the top half of the supernatant was isolated to ensure that the supernatant contained no contaminant of the pellet. The pellet was resuspended in the original volume with 1% sarkosyl in PBS. For the SAA, ⍺Syn monomer was filtered through a 100kDa filter (Amicon) to remove any oligomers. The SAA was performed in black clear bottom 96 well plates (Nunc, REF 265301) as quadruplicates. Each reaction contained one 2 mm glass bead and 100 μL 0.75 mg/mL ⍺Syn, 10 μM ThT, and 3.75% (v/v) supernatant or resuspended pellet fraction. The plate was incubated in a CLARIOstar Plus (BMG Labtech) at 37°C with cycles of 1 min 700 rpm shaking and 29 min rest.

### Size-exclusion chromatography (SEC)

Size-exclusion chromatography (SEC) was performed using an ÄKTAmicro system with an attached Tricorn 5/200 column (cytiva) packed with Superdex 200 (GE Healthcare). Protein separation was performed at 0.05 mL/min, and the column was equilibrated with 1% sarkosyl in PBS before each run. Data were analyzed using the Unicorn 5.3 software.

### Negative-stain transmission electron microscopy (ns-TEM)

Electron microscopy was performed at the EMBION facility (embion.au.dk), iNANO, Aarhus University. 3 μL of a diluted sample was added to a 400 mesh collodion (Sigma Aldrich) and carbon coated copper grid (Pelco) that had been glow discharged 45s at 25 mA and 39 mbar using an EasiGlow (Pelco). After a 30s incubation, the grid was blotted using a 85 mm filter paper grade 1 (lot. no. 10302, Whatman) and washed/stained 3x with 3 µL 2% uranyl formate (Polysciences Europe GmbH) with blotting steps between each washing/staining step and a final blotting and drying step. Micrographs were collected using a Tecnai Spirit TWIN transmission electron microscope (ThermoFisherScientific) operated at 120 kV using a TemCam F416 CMOS camera and EM-Menu software (Tvips).

## Results

**FIGURE 1:**
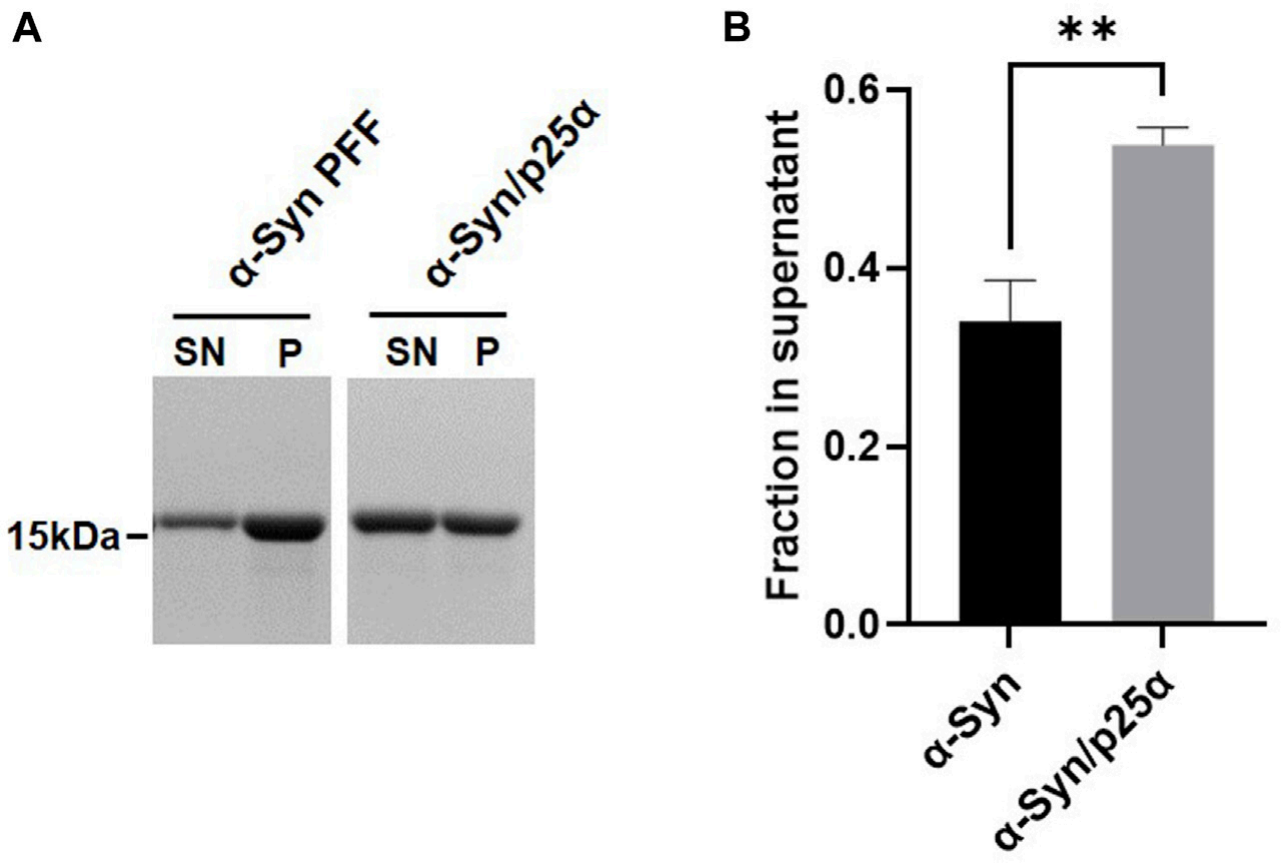
Different αSyn aggregate strains display differential stability toward sarkosyl treatment. The two synthetic strains, αSyn or αSyn/p25α PFF, were incubated with 1% sarkosyl in PBS for 30 min at RT. Soluble supernatant (SN) and insoluble pellet (P) fractions were isolated upon centrifugation. **(A)** Representative Coomassie Blue stained SDS–polyacrylamide gel of αSyn in SN and P fraction. **(B)** Images were quantified by densitometry as the relative amount of protein in the supernatant (SN/(SN+P)). Bars represent mean with one SD of three experiments. **p <0.01 on two-tailed unpaired t-test.

**FIGURE 2:**
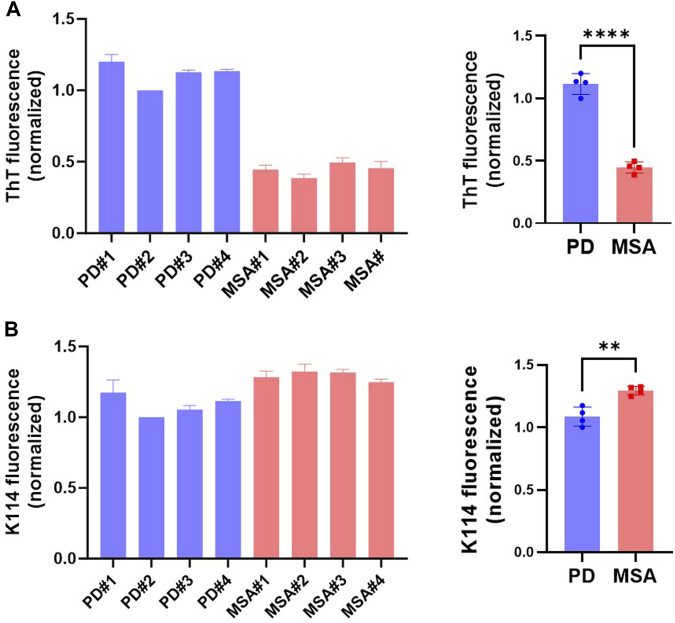
⍺Syn aggregate strains re-amplified from the seeds in CSF of patients with Parkinson’s Disease (PD) and Multiple System Atrophy (MSA) exhibit distinct fluorescence from amyloid-binding dyes K114 and ThT. Fluorescence emission of protein concentration adjusted patient-derived PFF from four PD and four MSA patients was measured with the fluorescent amyloid dyes **(A)** ThT and **(B)** K114. Y-axis represents arbitrary units normalized to the fluorescence of patient#2 PD PFFs. Left panel show measurements from the individual patient samples as mean of three replicate experiments. The right panel shows the four patient samples combined, with each data point representing one patient sample as a mean of three replicate experiments. Bars represented as mean with SD. **p <0.01; ****p <0.0001 on two-tailed unpaired t-test.

We hypothesize that αSyn strains exhibit differential stability in commonly used detergents, including sarkosyl. When studying ⍺Syn strain biology, this can have implications as some species may be lost during the extraction procedure or their amount underestimated. To test the hypothesis, we first compared the detergent stability of our *in vitro* generated prototype ⍺Syn strain αSyn/p25α, where aggregation was induced by the oligodendroglial protein p25α, to a *de novo* generated αSyn strain formed by spontaneous nucleation (Ferreira et al., 2021). Detergent instability was defined as a loss of insoluble filamentous αSyn in the pellet concomitant with its appearance in the soluble phase determined with a sedimentation assay. Incubating both strains with 1% sarkosyl for 30 min at RT solubilized 54% of αSyn/p25α filaments compared to 34% of the αSyn filaments (Figure 1). This demonstrates that strains-specific aggregate assemblies can exhibit different sensitivities to sarkosyl treatment. To extend our analysis to disease-relevant strains, we tested a small cohort of patient-derived ⍺Syn PFF from four Parkinson’s Disease (PD) patients (PD#1-PD#4) and four Multiple system atrophy (MSA) patients (MSA#1-MSA#4) that originally were amplified from patient CSF samples (Shahnawaz et al., 2020). The PD- and MSA-derived strains were characterized by their different fluorescence emission upon binding of two amyloid binding dyes, Thioflavin T (ThT) and K114. ThT emits significantly less when bound to MSA PFF than to PD PFFs (Figure 2; (Shahnawaz et al., 2020)), whereas K114 emits a slightly stronger signal when bound to MSA fibrils. This difference reflects a structural difference in the folding and packing of the β-pleated amyloid structure in the PD and MSA-derived strains.

**FIGURE 3:**
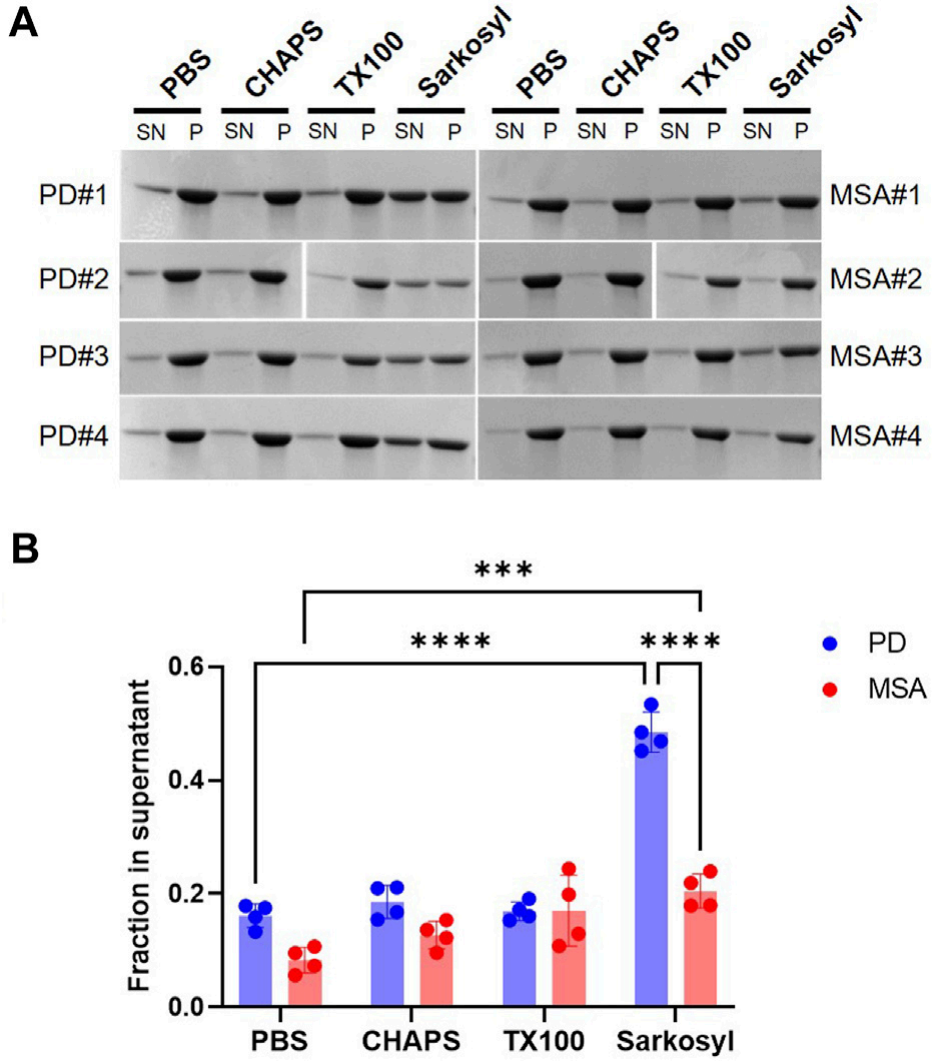
Stability of patient-derived PD- and MSA-aggregates in detergents. Four patient-derived PD- and MSA-PFF were incubated in PBS in the absence and presence of 1% CHAPS, 1% Triton X-100 (TX100), 1% sarkosyl for 30min, centrifuged, and separated in supernatant (SN) and pellet (P) fraction. **(A)** Representative Coomassie Blue stained SDS–polyacrylamide gels of SN and P fractions. **(B)** Images were quantified as the relative amount of protein in the supernatant (SN/SN+P). Each data point represents one patient sample as a mean of three replicate experiments, and bars represent mean and SD of four patient samples combined. Statistical analysis was made using one-way ANOVA and Tukey’s multiple comparison test. ***p <0.001; ****p <0.0001.

We tested the stability of the PD and MSA strains toward three commonly used detergents with different hydrophilic head groups. Zwitterionic CHAPS (1%), non-ionic Triton X-100 (1%), and anionic sarkosyl (1%). Sarkosyl solubilized more of the PD PFF (48%) compared to the MSA PFF (20%) (Figure 3). Neither 1% CHAPS nor 1% Triton X-100 affected the solubility of the MSA and PD PFF (Figure 3).

**FIGURE 4:**
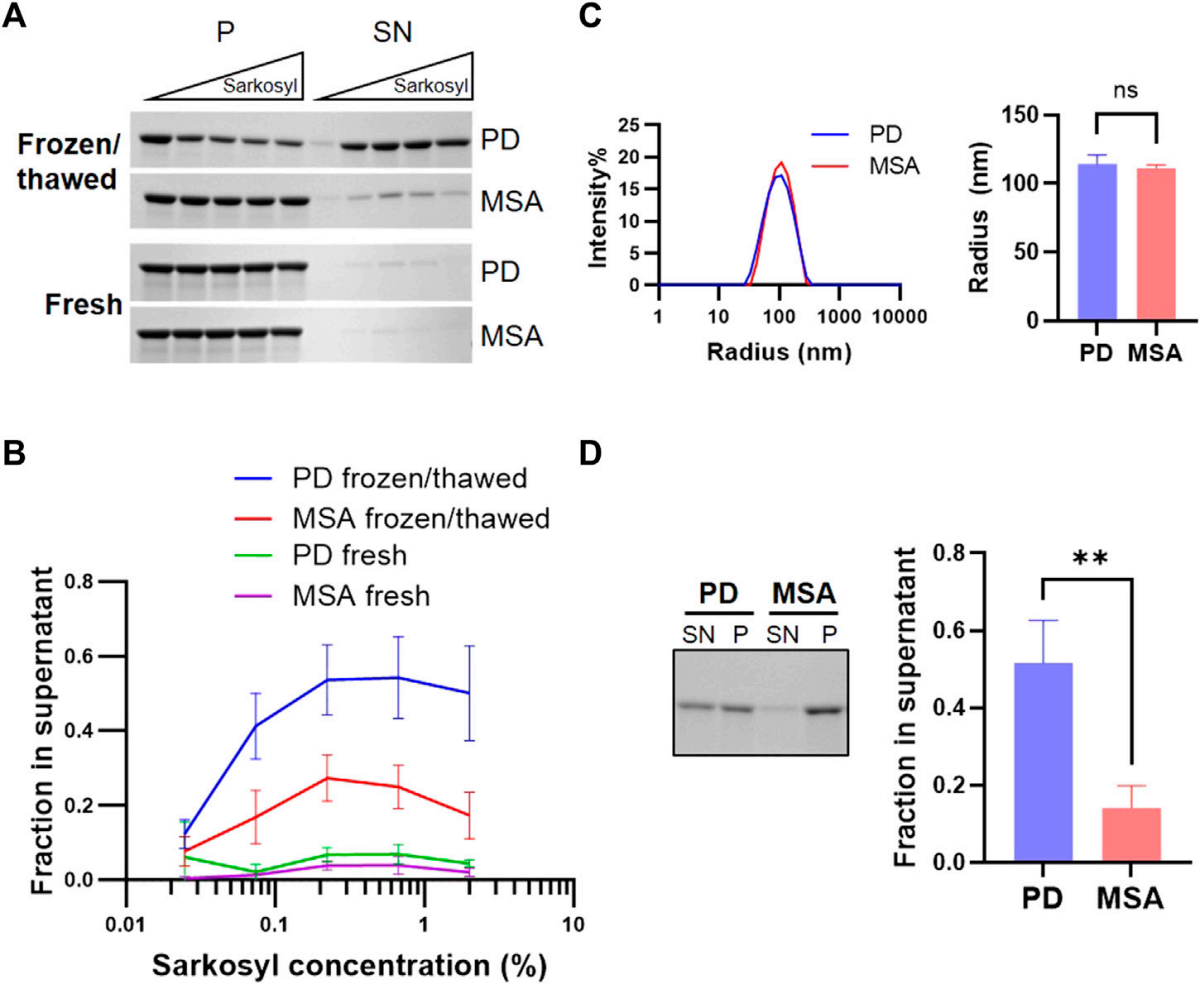
PD and MSA strain stability in sarkosyl after freezing/thawing and in sonicated aggregates. Freshly generated patient-derived PD and MSA PFF were either frozen at −80^o^C and thawed or kept at room temperature. **(A)** Upon thawing, the two pools of PFF were incubated with increasing concentrations of sarkosyl (0.025, 0.074, 0.222, 0.667, and 2%) for 30 min at RT and separated into supernatant (SN) or pellet (P) fractions by sedimentation assay, before being analyzed by SDS-PAGE. Representative Coomassie Blue stained SDS–polyacrylamide gels of SN and P fractions. **(B)** Quantification of the sedimentation assay represented as mean +/- SD from three experiments. Y-axis represents (SN/SN+P), and X-axis represents sarkosyl concentration. Note that a single freeze/thaw cycle increases sarkosyl sensitivity, and this effect is especially pronounced for PD PFF. **(C)** Dynamic light scattering (DLS) analysis of freshly prepared patient-derived PD and MSA fragments after sonication. The left panel shows scattering intensity on the Y-axis and the hydrodynamic radius on the log-scaled X-axis. The right panel displays the hydrodynamic radius of PD and MSA aggregate populations as mean and SD of three experiments. Non-significant (ns) based on two-tailed paired t-test. **(D)** Sonicated PD and MSA PFF were incubated with 1% sarkosyl or PBS for 30min, then centrifuged and separated in supernatant (SN) and pellet (P) fraction. The left panel demonstrates representative Coomassie Blue stained SDS–polyacrylamide gel of SN and P fractions. The right panel shows the quantification of (SN/SN+P). Note that sonication increases the sarkosyl sensitivity of the PD PFF, although both PD and MSA strains are broken into similarly sized filament fragments. Bars represent mean with SD from three experiments. **p <0.01 on two-tailed unpaired t-test.

Postmortem human brain tissue is often stored frozen in brain banks and storage conditions have been shown to influence the structural integrity of ⍺Syn PFF (Polinski et al., 2018). We investigated if a freeze/thaw cycle affected sarkosyl’s solubilizing effect on PD and MSA PFF. Freshly generated PD and MSA PFF were stored at either RT or frozen at −80 before being thawed for testing of the solubilizing effect of 0.075%–2% Sarkosyl. When freshly prepared, both PD and MSA PFF were resistant to 2% Sarkosyl. By contrast, one freeze/thaw cycle turned the PD PFF sensitive to sarkosyl, whereas the resistance of the MSA fibrils was less affected (approx. 50% versus 20%) (Figures 4A,B). The sensitivity to mechanical force from ultrasound waves was also tested. The sonication produced an almost identical fragment size for PD (114 nm) and MSA-derived PFF (111 nm) as measured by dynamic light scattering (DLS) (Figure 4C). Despite being broken into similar sized fragments, sonication increased the sarkosyl sensitivity of PD PFF to treatment to the same extend as freezing without affecting the MSA filaments (Figure 4D). Hence, physical treatment like freeze/thawing and sonication preferentially destabilize PD-derived ⍺Syn filaments to sarkosyl treatment, in contrast to MSA filaments.

**FIGURE 5:**
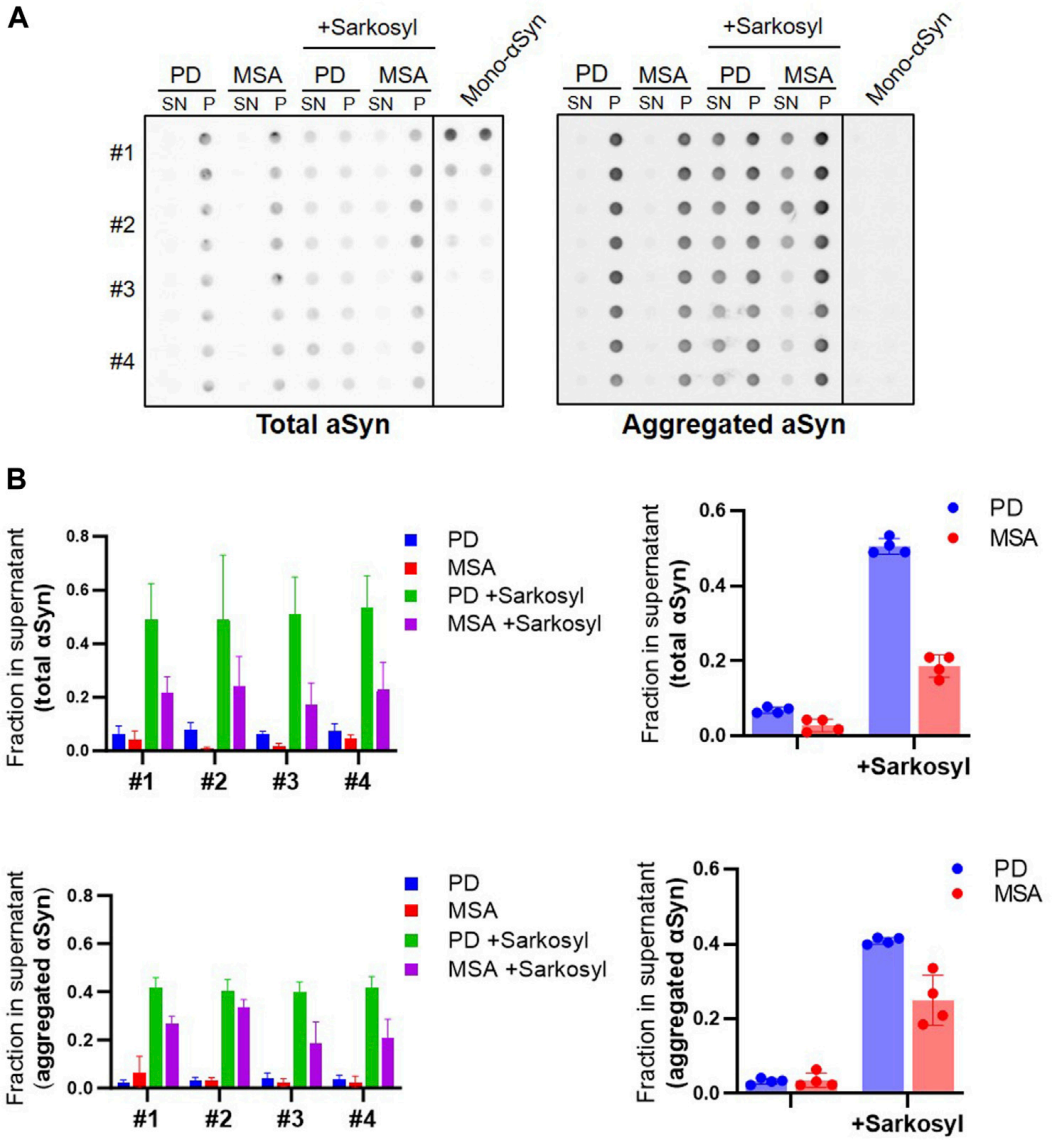
Sarkosyl treatment generates soluble aggregates of patient-derived PD and MSA PFF. Four patient-derived PD and MSA PFF were incubated with 1% sarkosyl or PBS for 30min, then centrifuged and separated in supernatant (SN) and pellet (P) fraction. **(A)** Fractions were applied in duplicates on a membrane and probed with antibodies for either total ⍺Syn (Syn1) or aggregated ⍺Syn (MJFR-14-6-4-2). Monomeric ⍺Syn (100 ng, 50 ng, 25 ng, 10 ng, and 5 ng) was applied on the membrane in duplicates. **(B)** Images were quantified as the relative amount of signal in the supernatant (SN/SN+P) upon subtraction of the background signal from each spot. Left panels show quantifications from the individual patient samples as mean of three replicate experiments. Right panels show the four patient samples combined, with each data point representing one patient sample as a mean of three replicate experiments. The experiment was repeated three times and each data point represents mean of the three technical replicates, and bars represent mean and SD of the four biological replicates.

We tested if the sarkosyl-solubilized αSyn from PFF retains aggregate-specific properties by testing their binding of an aggregate-specific antibody MJFR 14-6-4-2. Patient-derived PFF were treated with 1% sarkosyl and subjected to centrifugation. The pellet and supernatant fractions were analyzed by dot blotting (Figure 5A, left panel). The sarkosyl soluble fractions accounted for approx. 50% of the immunoreactivity from the PD PFF and approx. 20% from the MSA PFF as determined by the Syn-1 antibody binding to the dot blot (Figure 5B, right panel). We found a similar distribution with the aggregate-specific antibody MJFR 14-6-4-2 that did not bind to the monomer control sample (Figure 5A, right panel). This demonstrates that the Sarkosyl treatment does not only destabilize PFF into monomeric ⍺Syn, which does not bind the aggregate specific antibody (Figure 5A, right), but into soluble aggregated oligomeric species. We also noted a larger variation in the amount released as soluble aggregates from MSA patient samples, perhaps indicating a heterogeneity within the MSA disease strains (Figure 5B).

**FIGURE 6:**
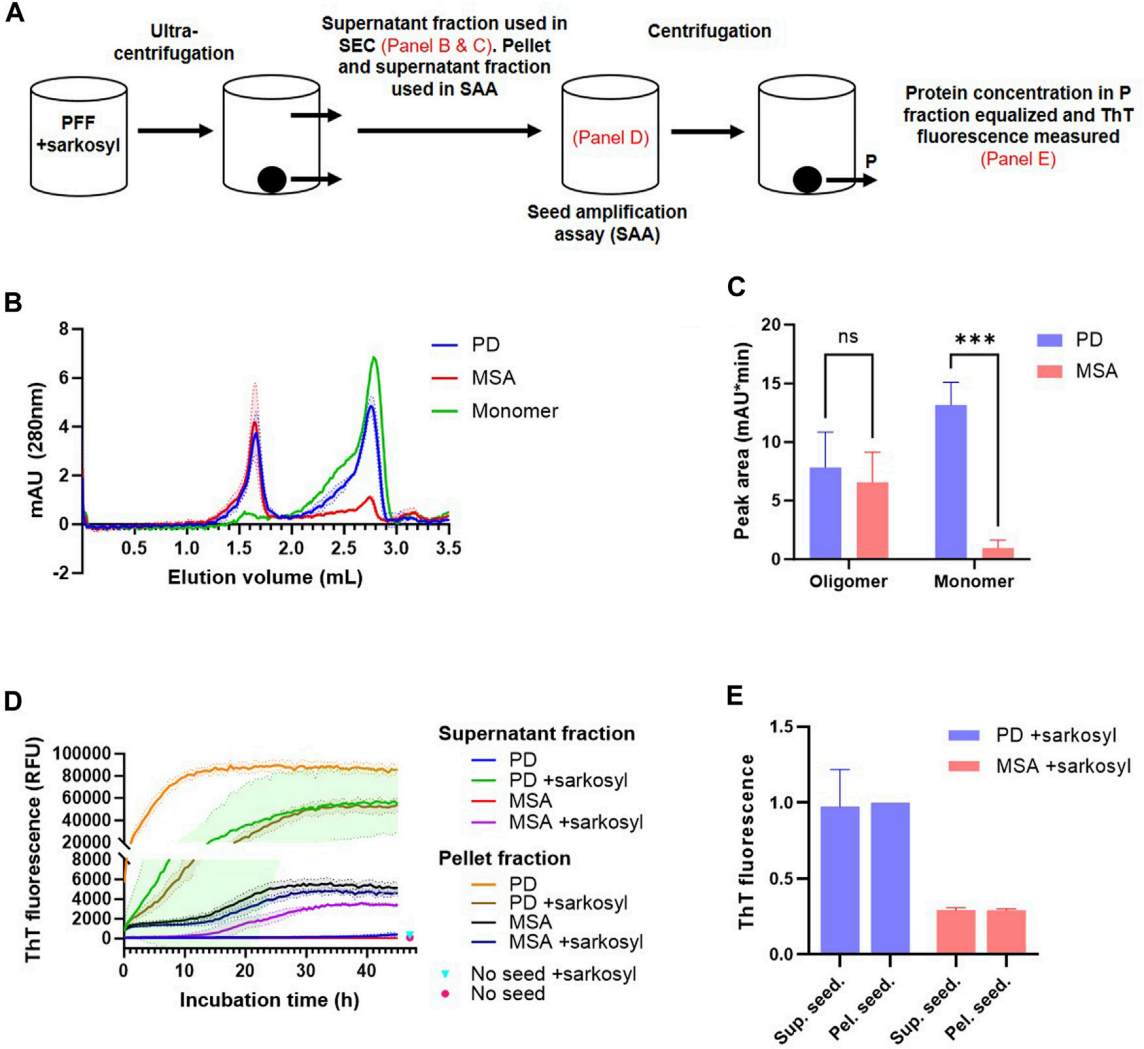
Sarkosyl solubilized patient-derived PFF species aggregates species are seeding competent and retain aggregate-strain specific characteristics. 1 mg/mL patient-derived PD and MSA PFF were incubated in the absence and presence of 1% sarkosyl in PBS for 30 min before separation into soluble supernatant (SN) and insoluble pellet fractions (P) by ultracentrifugation (300.000 x g, 1 h). **(A)** Overview of the experimental setup with annotations of what steps are illustrated in figure. **(B)** The supernatant fraction was analyzed by size-exclusion chromatography (SEC) using 1% sarkosyl in PBS as running buffer. Curves represent mean and SD of three experiments. Also depicted is the elution profile of an equivalent volume of 1 mg/mL monomeric ⍺Syn. **(C)** Quantification of the area under the curve of the two SEC peaks (oligomer peak 1.65 mL, monomer peak 2.75 mL). Bars represent mean with SD from three experiments. ***p <0.001 by two-way ANOVA with Sidak’s multiple comparisons test. **(D)** The pellet fraction was resuspended in the original volume with 1% sarkosyl in PBS, and both supernatant and pellet fraction were tested in a Seed Amplification Assay (SAA) at a final dilution of 3.75% (v/v) pellet and supernatant fraction versus monomeric ⍺Syn solution (0.75 mg/mL). The Y-axis is segmented to better illustrate all curves. Mean values from un-seeded control reactions at 47 h incubation time are illustrated as points. Curves represent mean with SD of three replicate experiments. **(E)** The reaction products of the SAA were isolated and centrifuged. The supernatant seeded (sup. seed.) and pellet seeded (pel. seed.) insoluble aggregates were resuspended in PBS and adjusted to 1 mg/mL. Thioflavin T fluorescence of the isolated aggregates was tested. Y-axis represents arbitrary units normalized to the fluorescence of PD+sarkosyl pel. seed. Bars represent mean with SD of three replicate experiments.

The nature of the solubilized fraction was analyzed by 1) size-exclusion chromatography, 2) negative staining-TEM, and 3) tested for their seeding competency, i.e., ability to seed aggregation of monomeric ⍺Syn, and if such seeded-PFF retain structural characteristics present in the parental insoluble PFF before sarkosyl treatment (Figure 6A). Centrifugation speed and time were increased to 300,000 x g for 1 h to ensure that no insoluble protein remained in the supernatant fraction. Figures 6B,C demonstrates that the sarkosyl soluble fraction of PD- and MSA-derived PFF contain equal amounts of high-molecular-weight species, while they differ in their amount of solubilized monomer. This indicates that the strain-specific detergent sensitivity is defined by a preferential solubilization of PD PFF into monomer. Negative staining-TEM of the soluble fraction was complicated by the large signal from the sarkosyl, but small fibrillar fragments could be detected in the supernatant fraction (Supplementary Figure S4). Seed amplification assay (SAA) was used to detect seeding competent species in the various fractions. Please note the segmented Y-axis to allow demonstrating the lower ThT emission from MSA PFF as compared to PD PFF (Figure 6D). Evidently, the soluble aggregates released by the sarkosyl treatment from both PD and MSA PFF are seeding competent as evidenced by a decreased time to obtain maximum ThT fluorescence in the SAA assay. Analyzing the insoluble fractions clearly demonstrates the seeding activity of the PD PFF are strongly reduced by the sarkosyl with T1/2 increased from about 1 h to > 10 h. This contrasts the insoluble MSA fractions that are essentially unaltered by sarkosyl (Figure 6D).

MSA PFF displays a lower ThT signal than PD PFF (Figure 2; (Shahnawaz et al., 2020)). We used this characteristic to test if the sarkosyl dissociated seeds retain structural characteristics from the parental PFF strain that they were released from. Figure 6E demonstrates that the insoluble material recovered in the SAA assay after 45 h when seeded with MSA material (adjusted to 1 mg/mL), displays low ThT signal, irrespectively of whether the reaction was seeded by sarkosyl-soluble or insoluble material. Likewise, is the PD material of high ThT signal irrespective of being seeded with sarkosyl soluble or insoluble material. Moreover, all fractions that reached a plateau in ThT fluorescence within 45 h contained predominantly insoluble ⍺Syn at end-point (Supplementary Figure S5). The only fractions that retained soluble ⍺Syn after 45 h incubation were those seeded with the soluble fraction of PD and MSA PFF that not were treated with sarkosyl, which corroborate the stable nature of the naïve ⍺Syn PFF. Hence, Sarkosyl solubilizes PD and MSA PFF with different efficiency, but the species being released contains aggregated oligomers that retain strain-specific seeding properties, and not just monomers.

## Discussion

Studies of brain-derived amyloid filaments have been of pivotal importance for our understanding of common neurodegenerative diseases, as exemplified by tau-filaments from Alzheimer’s disease (Guo et al., 2016; Shi et al., 2021). Solubilization of brain fractions in the detergent sarkosyl to isolate sarkosyl-insoluble filamentous species has been used as a common strategy to isolate the fibrils for structural and functional studies (Grazia Spillantini et al., 1998; Miake et al., 2002; Masuda-Suzukake et al., 2013; Schweighauser et al., 2020). We demonstrate that sarkosyl differentially degrades αSyn strains, both *in vitro* generated prototype strains and strains amplified from CSF of PD and MSA patients. This indicates that some αSyn filamentous species may be lost or underrepresented upon sarkosyl extraction and calls for consideration of whether other procedures could replace or supplement the standard sarkosyl extraction protocols.

The concept of differences in the stability of amyloid strains is not new, and aggregate strains of the prion protein exhibited different stability toward the chaotropic denaturant Guanidinium HCl, and the difference was hypothesized to be a factor in disease spreading (Safar et al., 1998). Likewise, αSyn extracts from MSA brain were less stable in Guanidinium HCl and induced a stronger pathology upon inoculation in a mouse model, whereas the more stable αSyn in extracts from PD and Alzheimer’s disease brains failed to induce pathology (Lau et al., 2020). By contrast, we demonstrate that the filamentous αSyn amplified from seeds in CSF from MSA patients are more stable in Sarkosyl than those amplified from seeds in PD. The relevance of this observation is corroborated by a study of the seeding activity of MSA brain-derived seeds that resisted up to 10% Sarkosyl before activity was lost (Woerman et al., 2018). This suggests that the solubility in the non-denaturing detergent sarkosyl relies on structural characteristics different from those affected by Guanidinium HCl. Such characteristics may well be determined by the folding of the N-terminal part of ⍺Syn, as fibrils generated from N-terminally truncated ⍺Syn (WT vs. ∆N10, ∆N20, ∆N30) differed in their solubility in 1% sarkosyl, where also an inverse correlation between sarkosyl stability and efficiency of *in vivo* propagation was reported (Terada et al., 2018).

Sarkosyl is a mild detergent that has been used extensively in protein purification, and amyloids have been considered insoluble in sarkosyl. Therefore, it has been widely used to extract amyloid-type proteins from brain tissue in general and ⍺Syn aggregates in particular. This strategy has successfully generated ⍺Syn filament strains whose structural analysis by cryoEM has yielded novel insights into ⍺Syn aggregates in brain (Schweighauser et al., 2020; Yang et al., 2022). Of note, two different structural ⍺Syn strains were demonstrated in the same MSA brain (Schweighauser et al., 2020). If subsets of brain-derived ⍺Syn aggregates exhibit different sensitivity to sarkosyl, as we show is possible for CSF-derived filaments, other populations may have been lost during extraction. The existence of multiple ⍺Syn aggregate strains within the same patient is supported by an SAA study of extracts from different brain regions affected by MSA (Martinez-Valbuena et al., 2022). Here the heterogeneity in SAA kinetics across brain regions in the PBS soluble fraction was lost when performing the parallel analysis on the sarkosyl insoluble fraction. This indicates that some of the complexities of aggregates are lost when only focusing on the sarkosyl insoluble fraction, especially because we demonstrate that sarkosyl treatment can convert filamentous ⍺Syn aggregates into both monomer and soluble aggregates, the latter with retained seeding activity.

Brains used for the purification of αSyn filaments have often been stored frozen in brain banks. Surprisingly, PD and MSA fibrils freshly prepared by seed amplification display equally low sensitivity toward sarkosyl. However, just a single round of freezing and thawing affects the physicochemical properties of PD filaments more than MSA fibrils making them less stable to sarkosyl treatment. The same sensitivity is also noted upon subjecting them to mechanical energy by sonication, further corroborating a less stable structure. Hence, freezing and sonication, two common elements in the workflow of processing brain tissue for αSyn aggregate extraction, can significantly affect the outcome if combined with sarkosyl extraction. Here, one may consider if other detergents, e.g., Chaps and Triton X-100, can be used as they do not destabilize the PD filaments in our study.

Sarkosyl disrupted the insoluble PD filament structure, so approximately 50% was turned into soluble species. Figure 6 demonstrates that these species were soluble after centrifugation at 300.000 x g for 1 h. We found that release of monomer accounts for the difference in stability between PD and MSA, and we found equal amounts of soluble aggregates in sarkosyl-treated PD and MSA based on gel filtration analysis. A recent study of brain-derived Abeta fibrils found small filaments can be pelleted by >250.000 x g centrifugation for 1 h (Stern et al., 2022). Although very few, we did observe fibrillary fragments in the sarkosyl-containing supernatant using negative staining-TEM. Whether other oligomeric species were present was undeterminable with the large sarkosyl aggregates present.

Conclusively, our results demonstrate αSyn aggregate strains exhibit different stability to sarkosyl treatment. A difference that first may become apparent upon physical processing of the brain tissue by, e.g., freezing and sonication. The destabilization converts the filaments into soluble seeding-competent aggregates that retain strain-specific characteristics from their parental filaments.

## Data Availability

The original contributions presented in the study are included in the article/Supplementary Material, further inquiries can be directed to the corresponding authors.

## Supplementary material

SUPPLEMENTARY FIGURE S1 Full scans of gels shown in Figure 3A.

SUPPLEMENTARY FIGURE S2 (A) Full scans of gels shown in Figure 4A. (B) Full scan of the gel shown in Figure 4D. The red box indicates relevant lanes.

SUPPLEMENTARY FIGURE S3 Full scans of gels shown in Supplementary Figure S5. The red box indicates relevant lanes.

SUPPLEMENTARY FIGURE S4 1 mg/mL patient-derived PD PFF were incubated in 1% sarkosyl in PBS for 30 min before separation into soluble supernatant and insoluble pellet fractions by ultracentrifugation (300.000 x g, 1 h). Input PFF, supernatant fraction, pellet fraction, and 1% sarkosyl in PBS were added to copper grids, stained with uranyl formate, and visualized by transmission electron microscopy. The diameter of the fragments was measured at 14.67 nm and 15.17 nm in the supernatant and pellet fractions, respectively. Note that a total of only three fibril fragments were found in the supernatant fraction. Red arrowheads indicate fibril fragments. Scalebar = 100 nm.

SUPPLEMENTARY FIGURE S5 (A) Figure 6C with an unmodified Y-axis. (B) The SAA reaction products from panel A were centrifuged (25,000 x g, 30 min) and separated into supernatant (SN) and pellet (P) fractions, then analyzed by SDS-PAGE and Coomassie staining. All samples that reached a plateau in ThT fluorescence contained predominantly insoluble ⍺Syn at end-point.
