# Supplementary figures and images for: Sarkosyl differentially solubilizes patient-derived alpha-synuclein fibril strains
Source: Front Mol Biosci. 2023 Aug 9;10:1177556. doi: 10.3389/fmolb.2023.1177556 (PMC10445646; doi:10.3389/fmolb.2023.1177556)

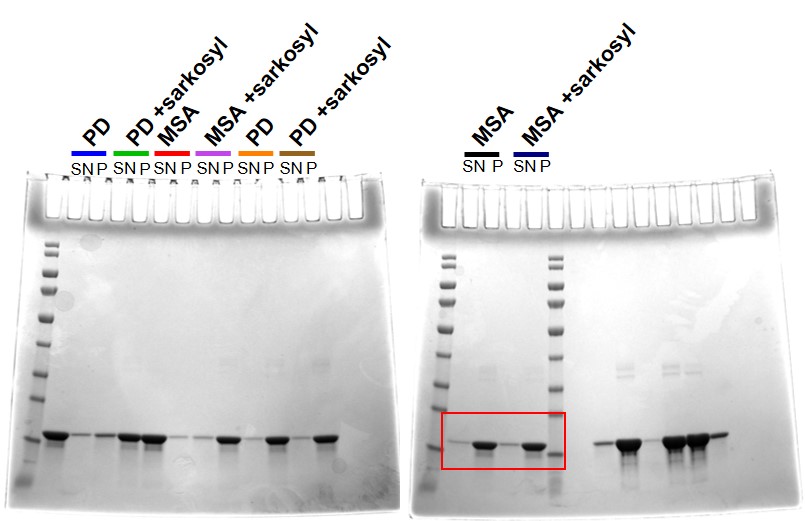

Supplement: Supplementary file 1 [file Image3.JPEG]

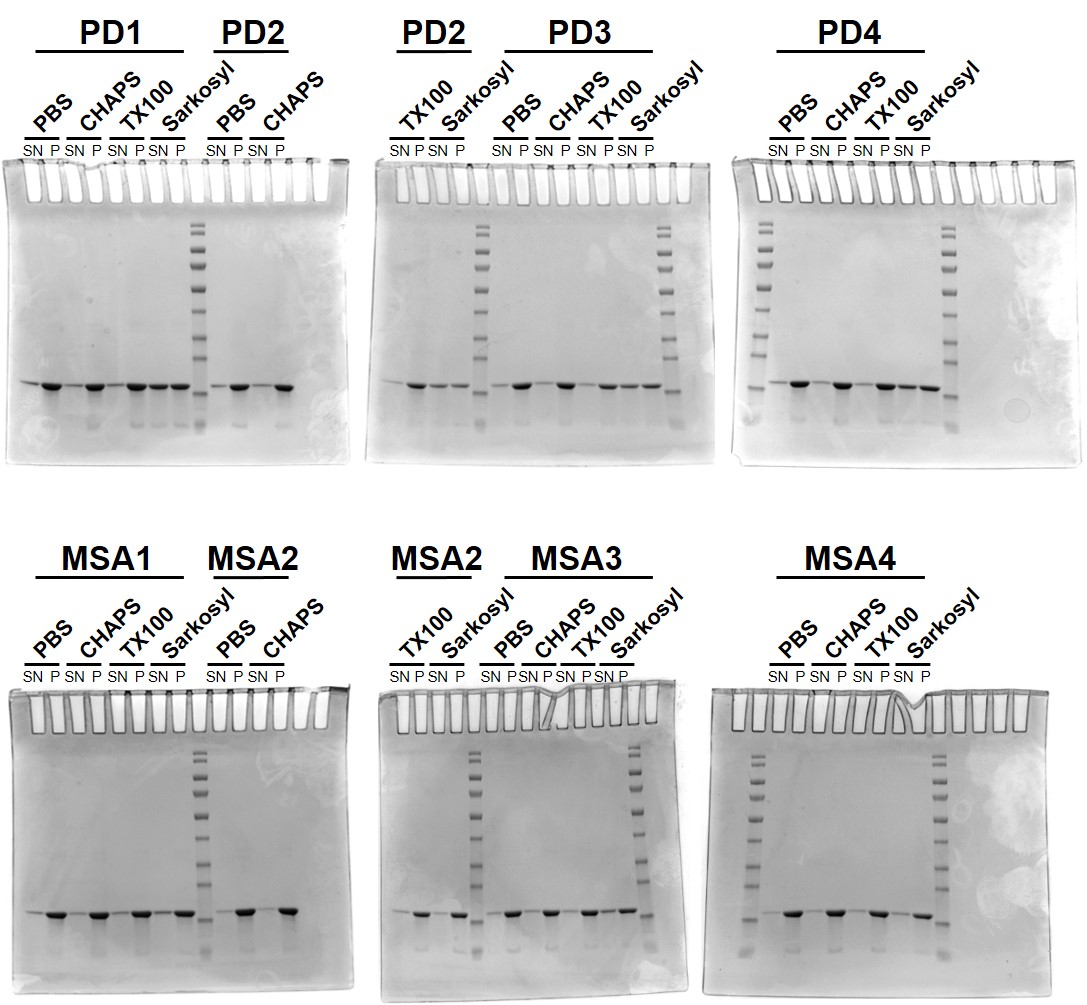

Supplement: Supplementary file 2 [file Image1.JPEG]

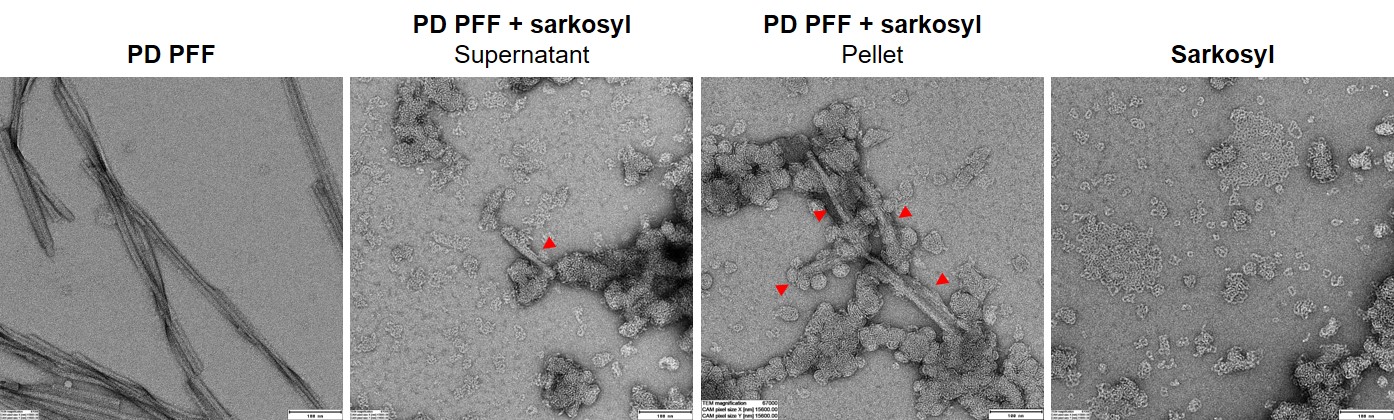

Supplement: Supplementary file 3 [file Image4.JPEG]

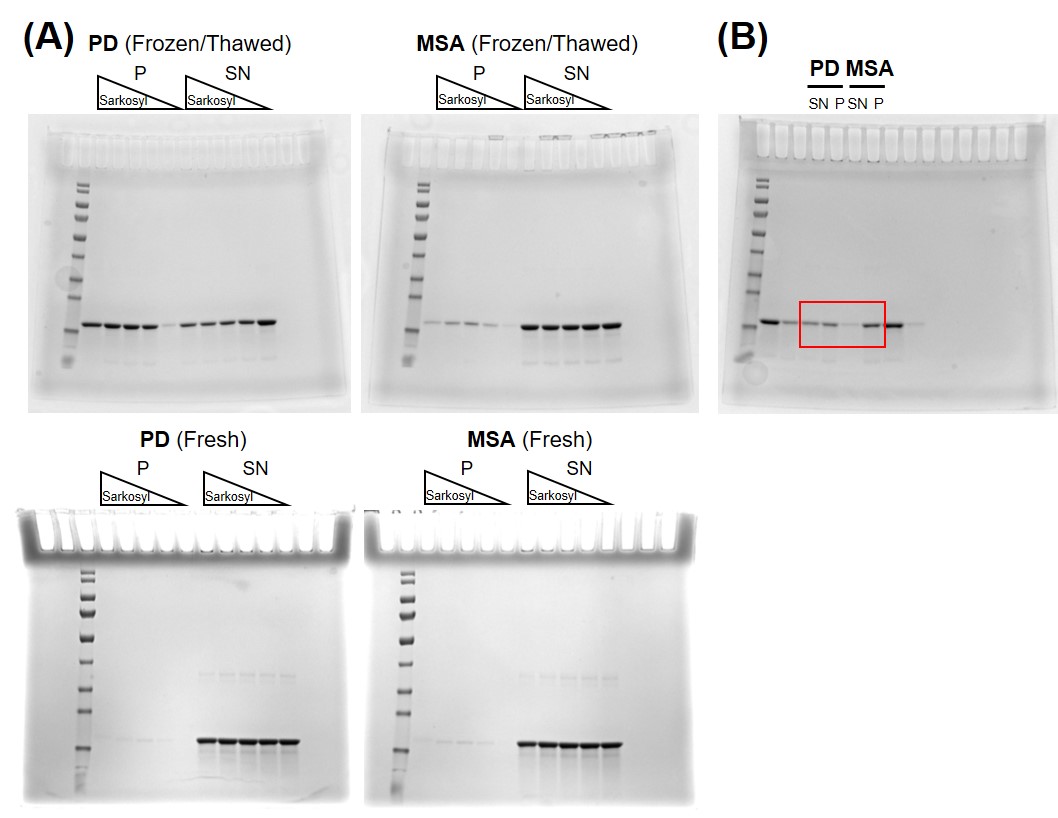

Supplement: Supplementary file 4 [file Image2.JPEG]

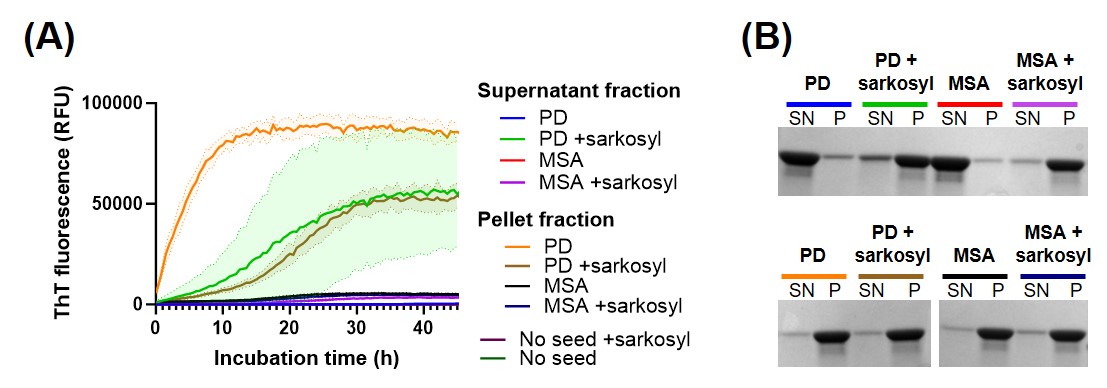

Supplement: Supplementary file 5 [file Image5.JPEG]
